# Supplementary material for: Integrated multiplexed assays of variant effect reveal determinants of catechol-O-methyltransferase gene expression
Source: Mol Syst Biol. 2024 Feb 14;20(5):2. doi: 10.1038/s44320-024-00018-9 (PMC11066095; doi:10.1038/s44320-024-00018-9)
Supplement: Supplementary file 1 — Appendix [file 44320_2024_18_MOESM1_ESM.docx]

**Appendix for “Integrated multiplexed assays of variant effect reveal determinants of catechol-*O*-methyltransferase gene expression”**

**Table of Contents**

Appendix Note

Untranslated regions influence variant effects on MB-COMT transgene expression (page 1)

Comment on RNA probing results for the *COMT* 3′ UTR (page 2)

Common population variants have no effect on MB-COMT transgene expression (page 2)

Comments on differences with May et al. 2023 study of yeast 5′ UTRs (page 3)

**Appendix Note**

Untranslated regions influence variant effects on MB-COMT transgene expression

Our analysis of ribosome profiling data for our MB-COMT transgene and endogenous TIS sites indicated uORFs in the 5′ UTR of *COMT* (**Figure 1C, Figure EV1B**). These uORFs are 74 and 28 nt upstream of the start codon for MB-COMT and are predicted to generate short peptides of 23 and 69 amino acids, respectively. However, because of our inclusion of a N-terminal Flag tag, uORF B no longer significantly overlaps the canonical MB-COMT ORF due to translation termination within the tag. To test whether these uORFs alter expression of COMT, we generated variants at the uORF A and B start codons (CUG>UUG); generated a silent variant in Flag that restores the endogenous uORF B frame; and deleted the Flag tag. Although we found no impact on protein abundance with the uORF A start codon variant, we found the uORF B start codon variant and Flag tag variants increased COMT protein abundance two to three-fold (two-sided Wilcoxon rank sum test p-value < 2.2 x 10^-16^, **Figure EV1C-E)**, suggesting uORF B represses translation of canonical MB-COMT. Additionally, deletion of poly-rC-binding protein (PCBP) motifs in the 5′ UTR led to a slight but significant increase in COMT protein abundance (~36% increase, Wilcoxon p-value < 2.2 x 10^-16^).

We serendipitously isolated a 53 nt deletion in the 5′ UTR of *COMT* during cloning that removed the putative uORFs (**Figure EV2A**). We assayed *COMT* mRNA abundance for the 5′ UTR deletion and 5′ UTR-containing control, and found the 5′ UTR deletion exhibited two-fold lower *COMT* mRNA abundance (**Figure EV2B**). We also ran polysome profiling experiments and determined that both the 5′ UTR-containing and 5′ UTR deletion mRNAs were highly loaded onto polysomes. Consistent with RT-qPCR readout, the 5′ UTR deletion showed reduced mRNA abundance in ribosomal subunits and the monosome to polysome fractions. Yet, we found no significant global shift in ribosome loading across polysomal fractions (**Figure EV2C**).

In contrast, the endogenous 5′ UTR had an inhibitory effect on protein abundance. By flow cytometry, the 5′ UTR deletion exhibited increased COMT protein abundance (**Figure EV2D**, two-fold increase in median fluorescence). Yet, mCherry fluorescence readout was similar between the 5′ UTR deletion and control, despite a modest difference reported by RT-qPCR. We suggest the discrepancy between mRNA abundance measurements by flow cytometry and RT-qPCR may be due to the long half-life of the mCherry protein [(Matreyek et al., 2020)](https://paperpile.com/c/KdLJjV/W9Xs). Yet, in agreement with flow cytometry results, Flag-immunoblotting reported 78% higher protein abundance for the 5′ UTR deletion (**Figure EV3C**).

We conclude that an element, or multiple elements, in the *COMT* 5′ UTR represses MB-COMT translation and reduces protein abundance. Our results are consistent with a mechanism in which uORF translation termination near the canonical start codon represses translation of MB-COMT within our transgene (**Figure EV2E, Discussion**). An alternative interpretation is that disruption of RNA secondary structure near the canonical TIS increases the rate of initiation [(Tsao et al., 2011)](https://paperpile.com/c/KdLJjV/Fri4).

Comment on RNA probing results for the *COMT* 3′ UTR

An alternative hypothesis for lack of signal in the middle of the 3′ UTR of NM_000754.4 by DMS-MaPseq and DIM-2P-seq mutational profiling methods is the expression of shorter alternative transcript isoforms (represented among Gencode transcripts). Because DIM-2P-seq employs RNase digestion followed by poly-A selection, the signal can only be achieved for a limited region upstream of the poly-A tail. Similarly, lack of signal in this same region by DMS-MaPseq may simply reflect predominant expression of isoforms with shorter 3′ UTRs.

Common population variants have no effect on MB-COMT transgene expression

Prior characterization of haplotypes comprised of the coding SNPs rs4680, rs4818, and rs4633, which form low-, average-, and high-pain sensitivity phenotypes (LPS, APS, HPS), indicated APS and HPS haplotypes decrease enzyme activity compared to the LPS haplotype [(Nackley et al., 2006)](https://paperpile.com/c/KdLJjV/MOkV). However, these effects were only observed when the haplotypes were expressed from a full-length *COMT* transcript with 5′ and 3′ UTRs [(Nackley et al., 2006;](https://paperpile.com/c/KdLJjV/MOkV) Supplementary Figure 6). In addition, the APS haplotype exhibited *increased* protein abundance in an *in vitro* context and in HEK293 and MCF-7 cells, but not COS-1 or HepG2 cells. Increased protein abundance for the APS haplotype was hypothesized to result from a less stable secondary structure at the TIS of S-COMT owing to the rs4633 C>T variant. These results suggested that expression phenotypes of the common haplotypes in *COMT* are cell-type specific and depend on either the 5′ UTR, 3′ UTR, or both UTRs.

We individually assayed five common population variants for effects on *COMT* RNA and protein abundance (LPS haplotype without rs6269): rs4680 (G>A), rs4818 (G>C), rs4633 (C>T); as well as rs6267 (G>T) and rs74745580 (C>T), which were previously associated with disease phenotypes or reported to alter *COMT* expression [(Gothelf et al., 2014; Lin et al., 2017; Li et al., 2014)](https://paperpile.com/c/KdLJjV/Itao+ZKim+EBf8) (**Figure EV3A**). These single variants enabled us to test effects for the partial HPS (rs4818) and LPS-T166 (rs4633) haplotypes (lacking rs6269), which led to low and high protein abundance, respectively [(Tsao et al., 2011)](https://paperpile.com/c/KdLJjV/Fri4). In our transgene, we found no difference in mRNA or protein abundance for any of the variants by flow cytometry or immunoblotting (**Figure EV3B-C**). Our results suggest that the prior observed effects of the common population variants may require expression from a native context (including endogenous UTRs).

Importantly, our study does not rule out a model where rs4633 facilitates translation initiation. Nevertheless, our data suggest a potential concurrent mechanism where rs4633 leads to higher protein abundance in human cell lines and in an *in vitro* translation assay (Tsao et al. 2011) by increasing RNA abundance. We note that Tsao et al did not directly measure RNA abundance in their study. In Supplementary Figure 3A of Nackley et al. 2006, the APS haplotype containing rs4633 C>T showed slightly higher total RNA abundance compared to the LPS haplotype (in our study, the wild-type template). However, this was not statistically significant and was only observed for the S-COMT isoform. It is possible that our observations are compatible with the conclusions in Tsao et al. 2011. For example, increased translation of rs4633 C>T may lead to stabilization of the RNA.

Comments on differences with May et al. 2023 study of yeast 5′ UTRs

While May et al. found a strong correlation between polysome load and protein abundance, our study found no such association. There are several potential reasons for this discrepancy. First, May et al. analyzed a library of 5′ UTRs as opposed to coding variants. Different 5′ UTRs may be expected to more significantly alter ribosome load on the reporter due to effects on translation initiation as opposed to translation elongation. That is supported by the much larger magnitude of effect sizes observed in May et al. for the 5′ UTRs compared to our library of single-codon changes, which have relatively small impacts on translation elongation and smaller effects on ribosome load. Thus, the correlation between ribosome load and protein abundance may become more apparent with larger effects on ribosome load.

Second, May et al. used an expression construct in which the reporter’s mCherry proxy for RNA abundance was unlinked from the YFP gene for reporting protein abundance. Conversely, our construct linked the mCherry (RNA proxy) and moxGFP (protein) reporters in a bicistronic transcript through use of an IRES. This means that our construct is likely to have higher polysome loading to begin with, which may challenge sensitivity to detect minor or modest variant effects on ribosome load (**Discussion**).

Lastly, the analysis strategies employed in our study differed from that of May et al. We compared variant abundance in polysome fractions to total RNA abundance, whereas May et al. compared abundance for a single polysome metafraction to a [40S, 60S, monosome] metafraction, excluding the cytoplasmic, “top” fraction. Additionally, our flow cytometry strategy sorted cells for a specific gate of moxGFP versus mCherry fluorescence, as opposed to sorting cells based on a ratio of the protein versus RNA reporters.
