# Supplementary material for: Integrated multiplexed assays of variant effect reveal determinants of catechol-O-methyltransferase gene expression
Source: Mol Syst Biol. 2024 Feb 14;20(5):2. doi: 10.1038/s44320-024-00018-9 (PMC11066095; doi:10.1038/s44320-024-00018-9)
Supplement: Supplementary file 10 — Expanded View Figures [file 44320_2024_18_MOESM10_ESM.pdf]

Expanded View Figures

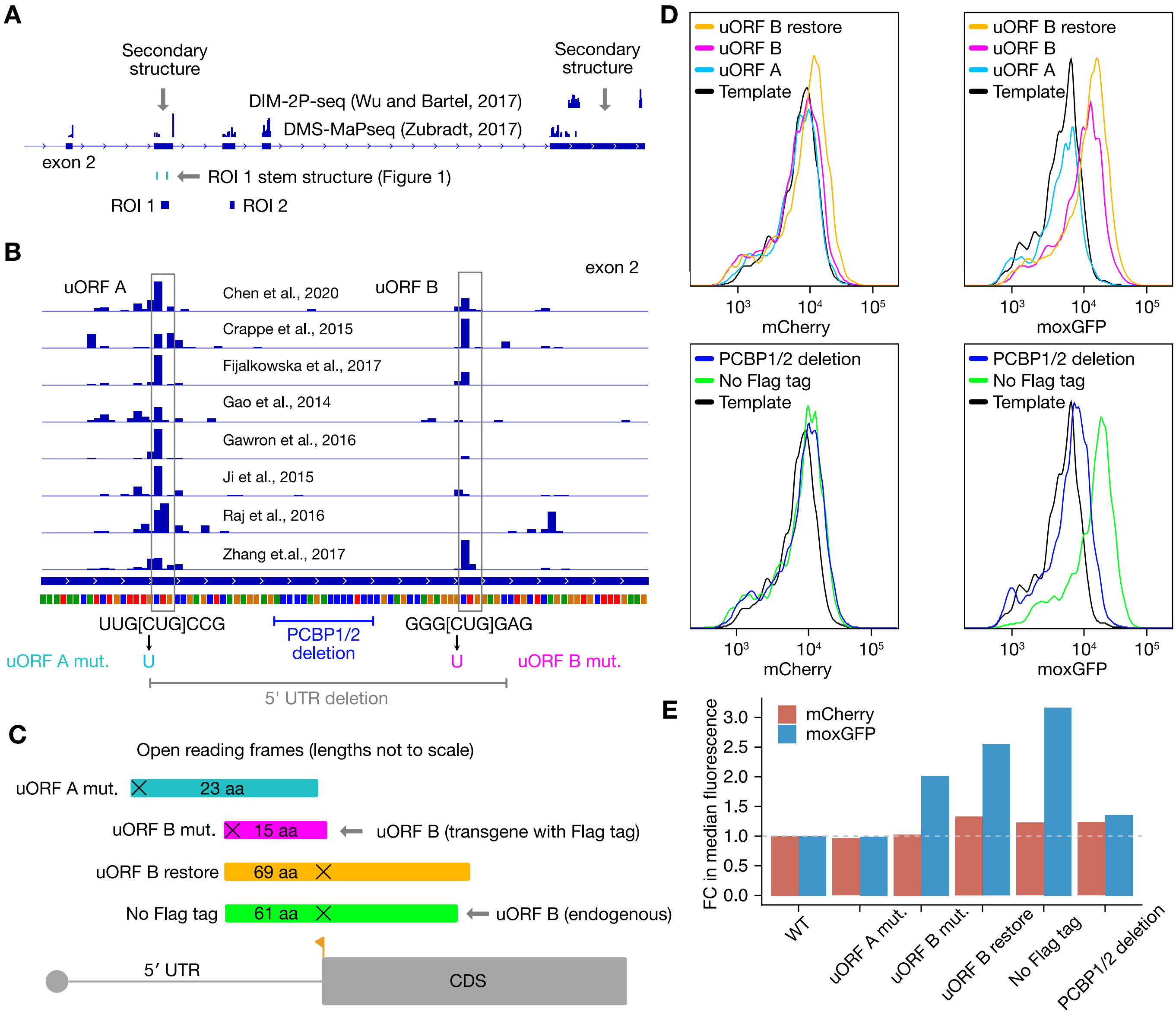

Figure EV1. ***COMT* 3′ UTR RNA secondary structure, uncharacterized uORFs, and noncoding variant effects on mRNA and protein abundance.**

(**A**) *COMT* RNA structure in the 3′ UTR. RNA probing data for HEK293T cells, available from the RASP database (Li et al, 2021), is shown. DIM-2P-seq employs polyA capture to enrich for structures at the 3′ end (Wu and Bartel, 2017). Gray arrows indicate sites of potential secondary structure. (**B**) *COMT* uORF translation initiation sites. TIS-ribosome profiling data from GWIPS-viz (Michel et al, 2014) was visualized. Two uORFs starting at CUG were identified in the 5′ untranslated exon 2 (NM_000754.4) in multiple cell lines including HEK293T. At bottom, isolated mutations and deletions are annotated, which are assayed in (**D**) and Fig. EV2 (5′ UTR del). (**C**) Schematic of uORF A and B reading frames. Black crosses indicate locations of the mutants. (**D**) Flow cytometry data of noncoding and Flag tag variants. See (**B**, **C**) for schematics. "uORF B restore" and "No Flag tag" variants restore a uORF B frame that overlaps the canonical ORF. "PCBP1/2 deletion" indicates deletion of poly(rC) binding protein (PCBP1, PCBP2) motifs. All variants with a reported fold change were significant by two-sided Wilcoxon rank sum tests at $p < 1 \times 10^{-9}$. (**E**) Fold change in median fluorescence for variants in (**D**). TIS translation initiation site, DMS dimethyl sulfate mutation profiling methods, uORF upstream open reading frame, UTR untranslated region.

© The Author(s)

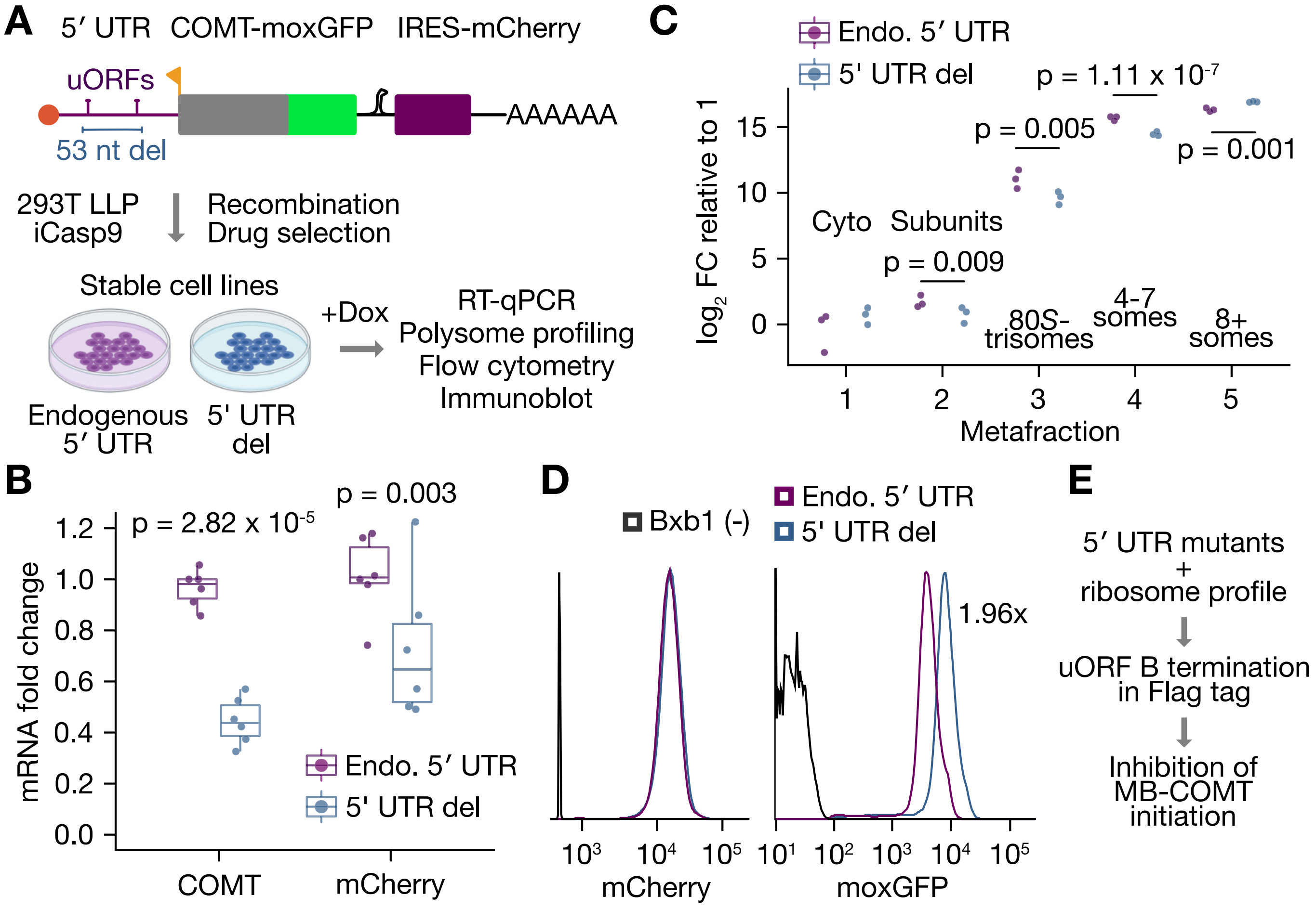

Figure EV2. The *COMT* 5′ UTR regulates mRNA and protein abundance.

In (**B**, **C**), significance was determined by two-sided Welch *t* tests. (**A**) Schematic of *COMT* transgene and strategy for stable expression. A *COMT* transgene with a partial 5′ UTR (exon 2 only) and a 53 nt deletion was cloned with a N-terminal Flag, C-terminal moxGFP, and bicistronic mCherry ORF. Transgenes were stably expressed in a HEK293T landing pad line and assayed following induction of transcription with Doxycycline (Dox). (**B**) The 5′ UTR del decreases *COMT* mRNA abundance. The mRNA fold change was determined by RT-qPCR and plotted relative to the median of Endo. 5′ UTR technical replicates ($N = 6$). The boxplot range is from the first to third quartile, center is the median, and whiskers extend to the largest and smallest values no greater than 1.5 * interquartile range. (**C**) The 5′ UTR del mRNA is decreased in low polysomes and enriched in high polysomes. RNA from raw polysome fractions were pooled into "metafractions" and RNA was extracted and assayed by RT-qPCR ($N = 3$ technical replicates). (**D**) The 5′ UTR del leads to higher COMT protein abundance. Flow cytometry results of the transgenic cell lines and a non-recombined (negative control) cell line are shown. (**E**) Proposed model for regulation by the 5′ UTR. 5′ UTR mutants indicated initiation at uORF B and termination within the Flag tag blocks initiation at the canonical TIS. FC fold change, TIS translation initiation site, moxGFP monomeric, oxidation-resistant GFP (Costantini et al, 2015), IRES internal ribosome entry site, 293T LLP iCasp9 lentiviral landing pad (Matreyek et al, 2020).

 © The Author(s)

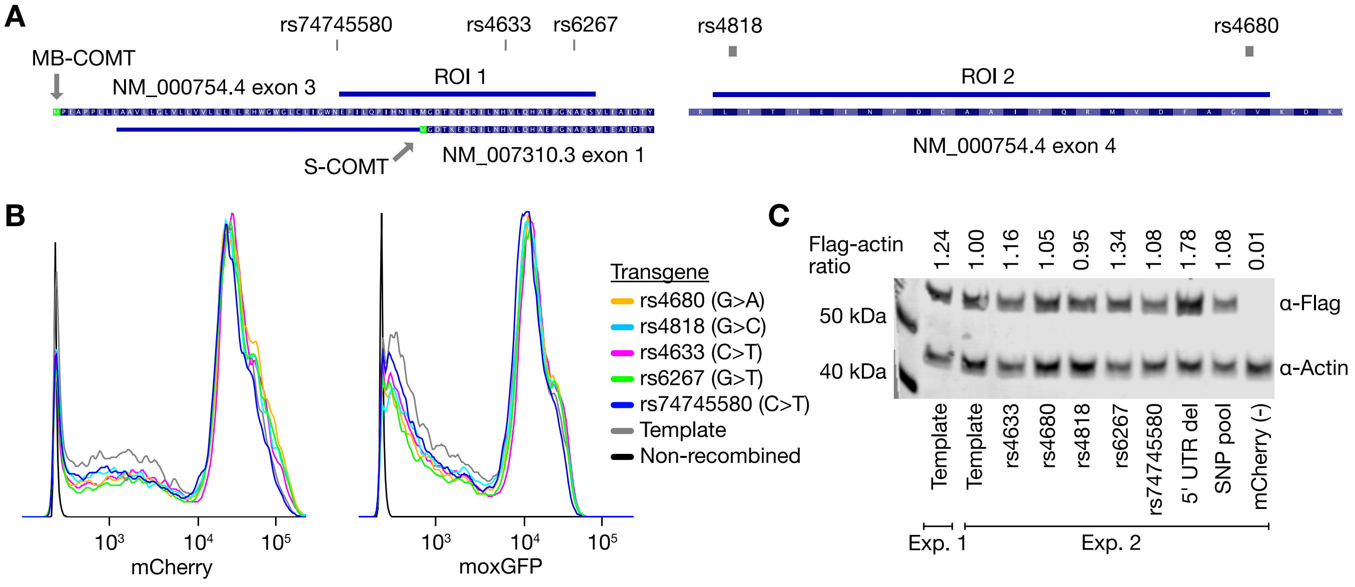

Figure EV3. ***COMT* common population SNPs have no impact on mRNA and protein abundance in the absence of complete UTRs.**

(**A**) *COMT* regions of interest and location of common population SNPs. (**B**) No impact of common population variants on *COMT* mRNA or protein abundance. Fluorescence of mCherry (mRNA proxy) and moxGFP (protein) is plotted for each common population variant (indicated with dbSNP identifiers), along with a non-recombined negative control and the non-mutagenized template, which consists of the LPS haplotype (Nackley et al, 2006) without the intronic SNP rs6269. (**C**) Immunoblotting of common population SNPs. Background-corrected Flag signal to Actin signal from densitometry analysis is indicated at the top of each lane. For the template transgene, lysates from two separate experiments were analyzed. "5′ UTR del" is the deletion mutant analyzed in Fig. EV2. "SNP pool" refers to a stable cell line with all common population variants. "mCherry (−)" refers to a negative control for Flag expression, an untagged mCherry payload.

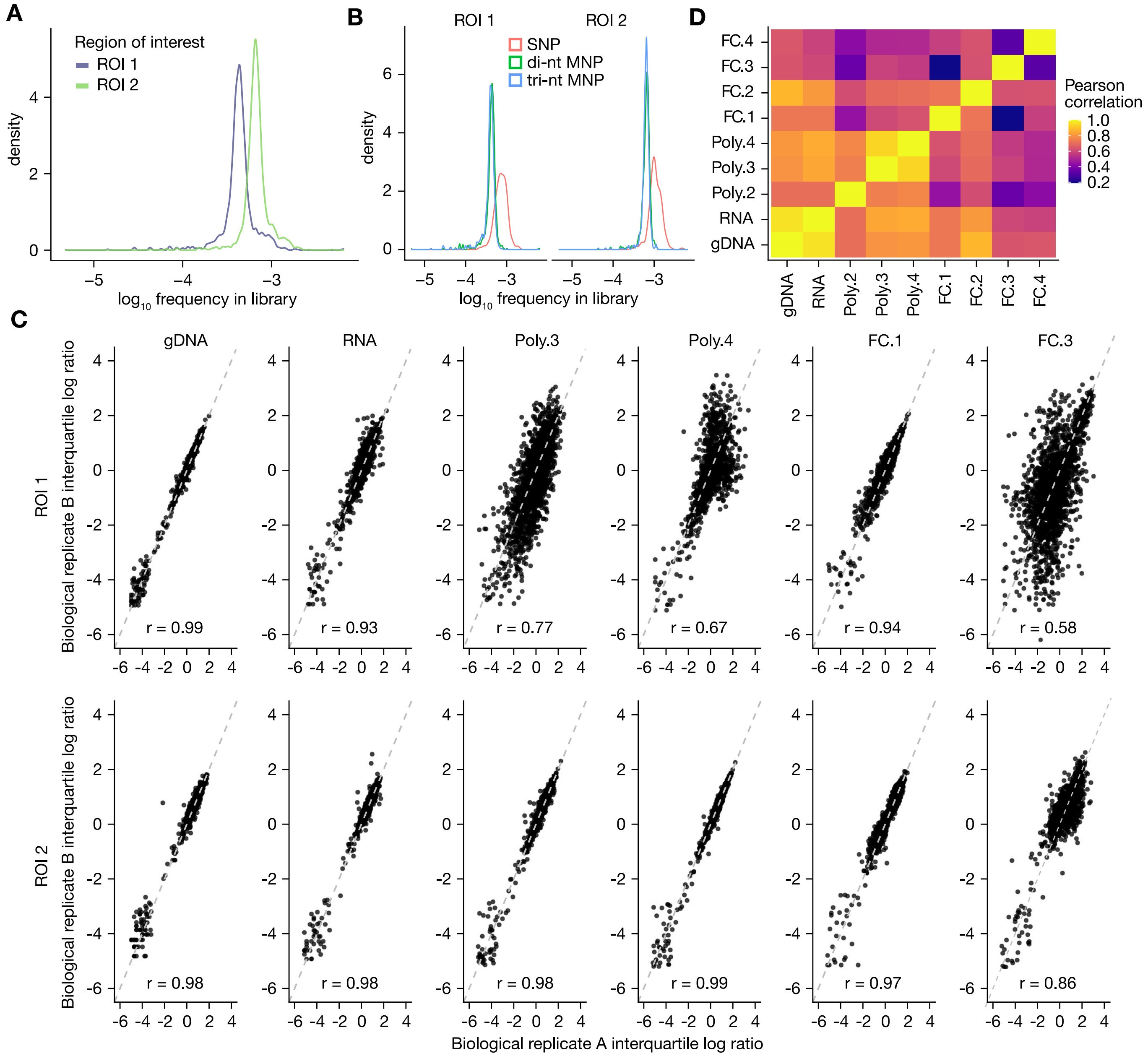

Figure EV4. *COMT* library quality control and reproducibility of variant abundance estimates.

In (**A**, **B**), plotted is kernel density estimation of $\log_{10}$ transformed frequencies reported in the Twist Biosciences quality control report. In panels C and D, and FC.1-4 refer to the four sorted flow cytometry populations, and Poly.2-4 refer to the second–fourth metafractions (see Fig. EV5D for schematic). (**A**) Variant uniformity for *COMT* target ROIs. (**B**) Frequency distributions by variant type. SNPs are at higher frequencies than di-nt and tri-nt MNPs, which have two and three mismatches, respectively. (**C**) Biological replicate Pearson correlation. Two of the four biological replicates are shown as examples. Metafractions and flow cytometry populations that were not subsequently analyzed (Poly.1, Poly.2, FC.2, FC.4) are omitted. (**D**) Correlation between gene expression readouts. Plotted is Pearson correlation of the median $\log_{10}$ frequency of biological replicates between readouts.

 © The Author(s)

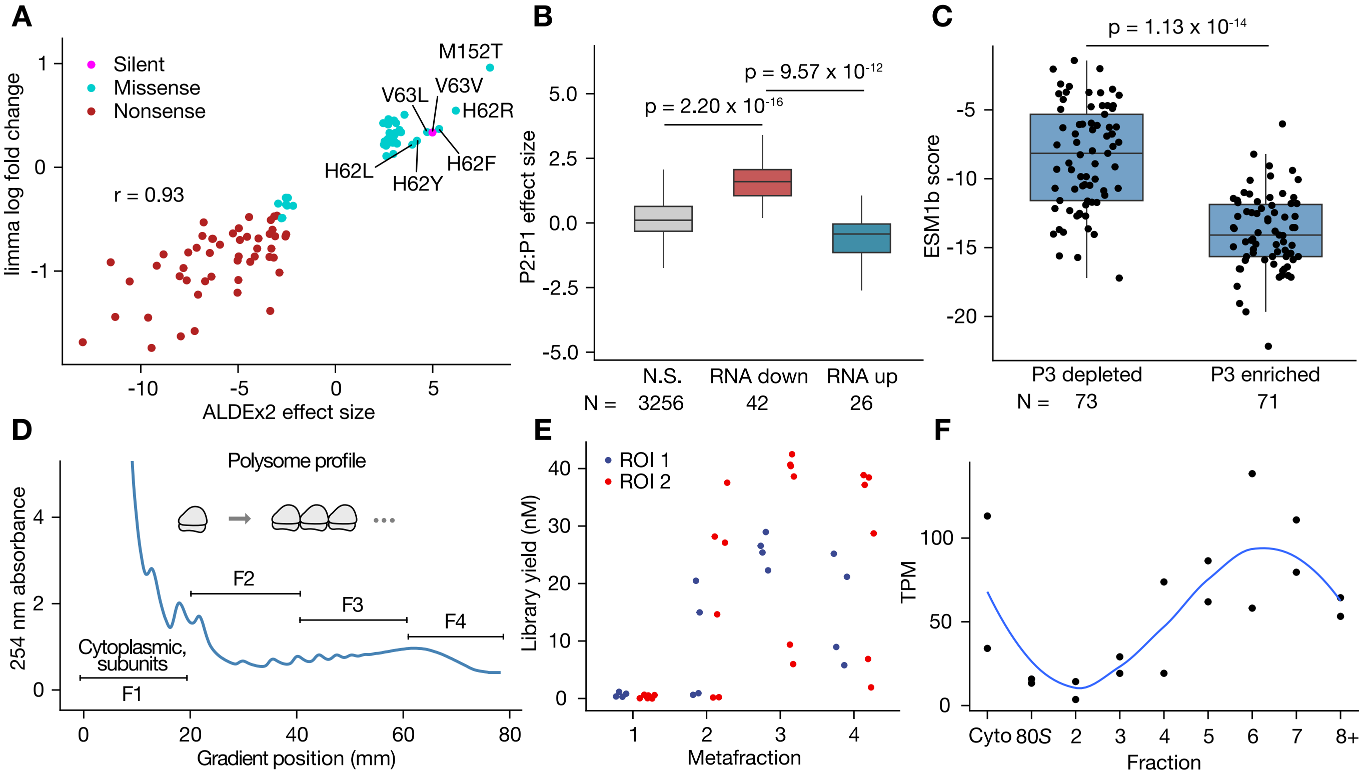

Figure EV5. Variant effect size measures, correlation of protein abundance effects, and low versus high polysome fractions.

In (**B**, **C**), significance was determined with two-sided Wilcoxon rank sum tests. The boxplot range is from the first to third quartile, center is the median, and whiskers extend to the largest and smallest values no greater than 1.5 * interquartile range. (**A**) Relation between limma and ALDEx2 effect measurements for RNA abundance. Pearson correlation coefficient is shown for variants at a FDR < 0.1. (**B**) Variants decreasing RNA abundance are enriched in flow cytometry P2 compared to P1. Outliers >1.5 * interquartile range were omitted for clarity. (**C**) Variants enriched in P3 have more negative ESM1b (Brandes et al, 2023) scores (log likelihood ratios) than those depleted from P3. (**D**) Representative polysome trace indicating the fraction pooling strategy. F1 and F2 were not subsequently analyzed due to inadequate library yield or reproducibility. (**E**) Yields for polysome metafraction libraries. Yield was quantified by qPCR using Illumina adapter specific primers ("Methods"). (**F**) Polysome profile of endogenous *COMT* in HEK293T cells. Transcripts per million mapped reads (TPM) is plotted using gene-level data from TrIP-seq (Floor and Doudna, 2016). Blue line is a loess fit with confidence intervals omitted for clarity. Cyto cytoplasmic fraction.

© The Author(s)
